# Transcriptional and Histochemical Signatures of Bone Marrow Mononuclear Cell-Mediated Resolution of Synovitis

**DOI:** 10.3389/fimmu.2021.734322

**Published:** 2021-12-08

**Authors:** Bruno C. Menarim, Hossam El-Sheikh Ali, Shavahn C. Loux, Kirsten E. Scoggin, Theodore S. Kalbfleisch, James N. MacLeod, Linda A. Dahlgren

**Affiliations:** ^1^Department of Large Animal Clinical Sciences, Virginia-Maryland College of Veterinary Medicine, Virginia Tech, Blacksburg, VA, United States; ^2^Gluck Equine Research Center, Department of Veterinary Sciences, College of Agricultural, Food and Environment, University of Kentucky, Lexington, KY, United States; ^3^Theriogenology Department, Faculty of Veterinary Medicine, Mansoura University, Mansoura, Egypt

**Keywords:** macrophage, arthritis, mevalonate pathway, oxidative stress, joint therapy

## Abstract

Osteoarthritis (OA) may result from impaired ability of synovial macrophages to resolve joint inflammation. Increasing macrophage counts in inflamed joints through injection with bone marrow mononuclear cells (BMNC) induces lasting resolution of synovial inflammation. To uncover mechanisms by which BMNC may affect resolution, in this study, differential transcriptional signatures of BMNC in response to normal (SF) and inflamed synovial fluid (ISF) were analyzed. We demonstrate the temporal behavior of co-expressed gene networks associated with traits from related *in vivo* and *in vitro* studies. We also identified activated and inhibited signaling pathways and upstream regulators, further determining their protein expression in the synovium of inflamed joints treated with BMNC or DPBS controls. BMNC responded to ISF with an early pro-inflammatory response characterized by a short spike in the expression of a NF-ƙB- and mitogen-related gene network. This response was associated with sustained increased expression of two gene networks comprising known drivers of resolution (*IL-10, IGF-1, PPARG*, isoprenoid biosynthesis). These networks were common to SF and ISF, but more highly expressed in ISF. Most highly activated pathways in ISF included the mevalonate pathway and PPAR-γ signaling, with pro-resolving functional annotations that improve mitochondrial metabolism and deactivate NF-ƙB signaling. Lower expression of mevalonate kinase and phospho-PPARγ in synovium from inflamed joints treated with BMNC, and equivalent IL-1β staining between BMNC- and DPBS-treated joints, associates with accomplished resolution in BMNC-treated joints and emphasize the intricate balance of pro- and anti-inflammatory mechanisms required for resolution. Combined, our data suggest that BMNC-mediated resolution is characterized by constitutively expressed homeostatic mechanisms, whose expression are enhanced following inflammatory stimulus. These mechanisms translate into macrophage proliferation optimizing their capacity to counteract inflammatory damage and improving their general and mitochondrial metabolism to endure oxidative stress while driving tissue repair. Such effect is largely achieved through the synthesis of several lipids that mediate recovery of homeostasis. Our study reveals candidate mechanisms by which BMNC provide lasting improvement in patients with OA and suggests further investigation on the effects of PPAR-γ signaling enhancement for the treatment of arthritic conditions.

## Introduction

Osteoarthritis (OA) is a common and debilitating condition that similarly affects horses and people (1, 2). Because chronic synovial inflammation is a hallmark of OA and often the single driver of related degenerative changes (3–7), the use of anti-inflammatory drugs (steroidal and non-steroidal) has been a logical and long-accepted approach for the treatment of many arthritic conditions (8, 9). However, acute inflammation is not simply a clinical sign to alleviate. Acute inflammation is a critical event in promoting tissue repair and setting the stage for endogenous resolution of the inflammatory process and recovery of homeostasis (10). Importantly, anti-inflammatory and pro-resolving effects are not the same, and resolution is not merely the passive termination of the inflammatory process. Anti-inflammation is based on inhibiting key pro-inflammatory mediators, such as chemokine and cytokine production and leukocyte extravasation to the site of injury. Resolution is an active process driven primarily by macrophages and their derived cytokines and lipid mediators, which shift the phlogistic phase of inflammation into a non-phlogistic process that culminates with tissue repair and recovery of homeostasis (11, 12). Most importantly, the recruitment of macrophages and the production of pro-resolving mediators is triggered by enzymes synthesized during the acute inflammatory process (13). Macrophages play such a fundamental role in resolving inflammation and promoting tissue repair that impaired macrophage chemotaxis and/or macrophage depletion results in inefficient healing or chronic inflammation (14–16). Blocking acute inflammation with anti-inflammatory medications interferes, at least to some degree, with macrophage recruitment and the pro-resolving response, and often prevents effective resolution and recovery of homeostasis (12, 13). Targeted therapies for chronic joint inflammation should therefore have pro-resolving properties, which precisely combine pro- and anti-inflammatory mechanisms (12).

Synovial macrophages are the central drivers of the inflammatory response in osteoarthritic synovium (17, 18). In fact, synovial macrophage activation is directly related to disease activity, severity, and pain in OA-affected patients (19). However, this relationship is not causative. Synovial macrophages are also essential keepers of synovial homeostasis through phagocytic clearance and secretion of anti-inflammatory and pro-resolving cytokines, chemokines, enzymes, and growth factors (20–23). Following injury, synovial macrophages proliferate to form a protective immunological barrier in the synovial lining for intra-articular structures (24, 25). When regulatory functions are overwhelmed by the amount of damage, synovial macrophages upregulate inflammation, signaling to monocytes and other leukocytes (e.g., neutrophils and lymphocytes) to help counteract the increased demands for tissue repair and restore homeostasis (17, 26). During the progression of OA, the recruitment of myeloid monocytes into joints seems to be impaired (27), which combined with continuous joint damage, overwhelms the pro-resolving mechanisms of synovial macrophages, leading to degeneration (17, 27, 28).

The mononuclear cell fraction of bone marrow aspirates (bone marrow mononuclear cells -BMNC) is a rich source of pro-resolving macrophages that have been used therapeutically to improve tissue repair and inflammation resolution (29–39). Macrophages within BMNC are the main drivers of such effect, which’s documented pro-resolving functions include increased production of IL-10 (30, 33), diverse types of prostaglandins and specialized lipid mediators (13, 40, 41). The production of these molecules induce decreased production of IL-6 (33), increased phagocytic clearance of debris and apoptotic cells (efferocytosis) (30, 39) and enhanced PPAR-gamma signaling (42–47). Increasing the numbers of myeloid macrophages present in osteoarthritic knees, by injection of BMNC, restored joint homeostasis with long-lasting effects (48). Similarly, BMNC therapy increased counts of pro-resolving macrophages and induced marked resolution of joint inflammation in *in vivo* and *in vitro* models of equine synovitis (49, 50). In these models, there was a coordinated spectrum of pro-inflammatory, pro-resolving and anti-inflammatory events, including increased IL-10, IGF-1, and PGE_2_ production, and self-limiting IL-1 signaling (α and β). These events are all innately required for efficient synovial homeostasis and tissue repair and are commonly antagonized by therapeutic corticosteroids (30, 51–53). While these findings partially explain the durable effects of BMNC in the treatment of OA, little is known about BMNC-related mechanisms of resolution. Therefore, our purpose was to identify cellular mechanisms from BMNC driving joint homeostasis that could be used for developing targeted pro-resolving joint therapies and uncovering biomarkers of arthritis resolution. The aim of this study was to identify transcriptional signatures of BMNC leading to inflammation resolution using RNA-sequencing and relate these to the expression of key gene products in the synovial membrane. We hypothesized that gene networks linked to macrophage proliferation, negative regulation of inflammatory response, and to a lesser extent, NF-ƙB signaling, would be temporally upregulated in response to inflammation.

## Materials and Methods

### Study Design

**Figure 1:**
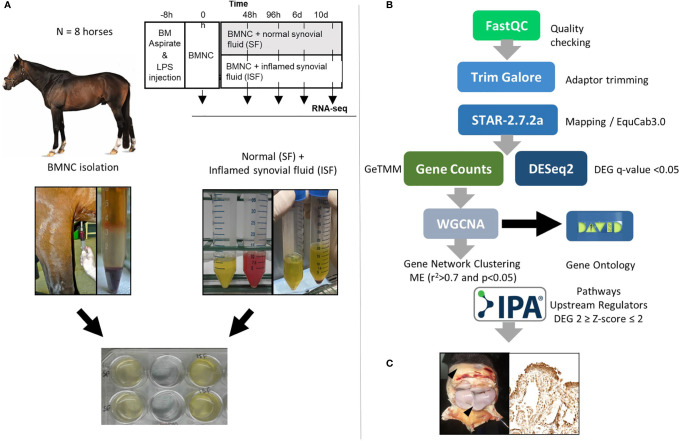
**(A)** Study design showing timing of bone marrow aspiration and induction of synovitis (LPS, 0.5 ng/joint) (top). Harvesting and processing of bone marrow for BMNC isolation, and normal (SF) and inflamed synovial fluid (ISF) (middle) for cell culture following depletion of native SF cells (bottom). **(B)** Bioinformatics pipeline used for RNA-sequencing, quantification and analysis. **(C)** Immunohistochemical assessment of the synovium of inflamed joints treated with BMNC leading to inflammation resolution or DPBS as a control for the expression of genes identified as upstream regulators or key in activated pathways.

Samples used in the current report were obtained from two previous studies using the same horses. These *in vitro* (50) and *in vivo* (49) studies were counterparts of a larger project assessing the effects of BMNC on joint inflammation resolution. Briefly, eight skeletally mature Thoroughbred horses (3-9 years old, median 5 years; 2 females and 6 castrated males) free of OA or systemic inflammation were used under IACUC approval and oversight. General and musculoskeletal health were confirmed by clinical, hematological and orthopedic evaluations. Following sternal bone marrow aspiration for BMNC isolation, synovitis was induced in both radiocarpal joints, as a way of producing more homogeneous inflammation and inflamed synovial fluid (ISF) than could be acquired from naturally occurring OA. Normal synovial fluid (SF) was collected from healthy middle carpal joints. BMNC from each horse were cultured independently (not pooled) in neat (100%) autologous SF or ISF and harvested at 0 (uncultured), 48 and 96 hours, and 6 and 10 days for RNA isolation. RNA-seq was used to identify transcriptional signatures of BMNC in response to acute joint inflammation. The transcriptome of BMNC was assessed over time within the same group (SF or ISF), as well as comparatively between groups at each time point (**Figure 1**). The expression of 9 potential upstream regulator genes identified following bioinformatical analysis was assessed by immunohistochemistry in the synovium of inflamed joints from the same horses, 6 days after treatment with BMNC or Dulbecco’s phosphate buffered saline (DPBS). Four protein coded by genes identified in key activated pathways were also assessed by IHC.

### BMNC Isolation, Induction of Synovitis, and Synovial Fluid Harvest

Bone marrow harvest and processing of BMNC, and induction of the synovitis model were performed as previously described in our related study (49). Briefly, BMNC were isolated by density gradient centrifugation. Synovitis was induced by intra-articular injection of 0.5 ng lipopolysaccharide (LPS) into each radiocarpal joint (49, 54). At peak inflammation (8 hours following induction of the model), SF and ISF were collected using aseptic technique. Synovial fluid cytology (SF and ISF) was performed to confirm the health of normal joints and ensure LPS effectively induced synovitis. Synovial fluid was then centrifuged (5,000g; 20 min; 4°C) for cell depletion and the cell-free supernatant used as autologous growth medium. Parameters used to differentiate SF from ISF included quantification of cytokine as reported in our related study (50), total protein (<2.5 g/dL in SF and >4g/dL in ISF) and synovial fluid cytology (total nucleated cells/µL < 1,500 in SF and ~130,000 in ISF; neutrophil count <10% in SF and >80% in ISF).

### BMNC Culture in SF and ISF

BMNC were plated in 24 well culture plates (2x10^6^ viable cells/50 µL DPBS/well) and covered with 500 µL SF or ISF. Cell viability was assessed at baseline using trypan blue and ranged from 74-96% across horses. Well contents were carefully mixed and plates incubated at 37°C in 5% CO_2_ and 90% humidity. Remaining SF and ISF was preserved at 4°C for later addition of medium to replenish cell nutrients (200 µL added every 48 hours). All conditions and time points were performed in duplicate wells with cells from one well used for RNA-sequencing and the other for flow cytometry (macrophage activation markers CD14, CD86, CD206 and IL-10). Conditioned medium was aspirated at the same time points of cell harvest (48 and 96 hours and 6 and 10 days) centrifuged, and the cell-free supernatant used for cytokine and growth factor quantification (FGF-2, GM-CSF, IL-1β, IL-6, MCP-1, IL-10, TNF-α, SDF-1, IGF- 1, IL-1ra, and PGE_2_) using a PGE_2_ ELISA kit (KGE004B; R&D Systems) and the Milliplex Map Equine chemokine/cytokine bead based array (Eqcttmag-93K,; MilliporeSigma). Details and findings from flow cytometry and cytokine and growth factor quantification are reported elsewhere (50) and were used in this study as a trait for weighted gene co-relation network analysis (WGCNA).

### Transcriptome Analysis of BMNC Cultured in SF and ISF

#### RNA Isolation and Sequencing

Cultured cells were recovered in 10 mM EDTA, centrifuged (12,000g; 10 min; 4°C) and the cell pellet placed in guanidinium chloride-phenol (Trizol^®^, Life Technologies, 15596018, Carlsbad, CA). RNA was purified with on-column DNase digest (DirectZol™ RNA microprep kit, R2061, Zymo Research, Irvine, CA), quantified (Qubit^®^ 3.0 Fluorometer, 33216, ThermoFisher Scientific, Carlsbad, CA), and stored at -80°C. RNA quality was assessed (Bioanalyzer 2100, Agilent Technologies, Santa Clara, CA) and cDNA libraries prepared using TruSeq DNA Library Preparation kits (Illumina, Inc., San Diego, CA), followed by sequencing (NovaSeq 6000 S4, Illumina) to generate an average of 34.5 (range, 24-54) million stranded paired-end reads (2 x 150 nt) per sample.

#### Bioinformatics Pipeline

Reads were trimmed for quality and adapters with TrimGalore 0.4.3 and mapped to the equine reference genome (EquCab 3.0) (55) using STAR (56) algorithm (version 2.7.2a) and GeneCounts, and expression values determined as gene length corrected trimmed mean of M-values (GeTMM) (57) with the Ensembl v104 annotation. Differentially expressed genes (DEGs) were determined using DESeq2 based upon a false discovery rate (FDR) adjusted *P*-value (q-value) <0.05 after Benjamini–Hochberg correction for multiple testing, by comparing datasets from consecutive time points within SF or ISF, and by comparing ISF to SF datasets at any given time point. The cutoff set for considering a transcript expressed prior to analysis was 10 fragment alignments. DEGs were represented by principal component analysis using JMP Pro 13 and by volcano plots using Origin software (version 2019, OriginLab, Northampton, MA, USA).

#### Functional Genomics

We adopted a multidisciplinary approach to functional genomics by employing several bioinformatics tools to tease out the biological significance of our data. We used WGCNA and DAVID in a semi-supervised analysis to identify biological processes of interest, and IPA to identify upstream regulators and activated and inhibited signaling pathways. By using this approach, we took advantage of both the superior annotation of biological processes from DAVID and the better pathway annotation of IPA. Together, these tools enabled us to make associations to our previous clinical studies to start to draw clinical translations to our findings.

Weighted gene co-relation analysis was performed using WGCNA version 1.66 package in R to construct gene co-expression networks as described elsewhere (58, 59). Gene co-expression clusters were generated from the whole transcriptome in SF and ISF datasets separately over time. Only genes expressed in at least 50% of samples in each dataset were included in the analysis (16,318 genes in SF and 18,038 genes in ISF). In order to normalize the data, the GeTMM values for each gene were log2 transformed. Next, a pairwise correlation matrix was constructed between all pairs of genes across the samples, and a matrix of weighted adjacency was generated by raising co-expression to a power β = 9, as determined for our sample set (58, 60). A topological overlap matrix (TOM) was then assembled and used as input for hierarchical clustering analysis. Then, a dynamic tree cutting algorithm was used to identify gene clusters or modules (i.e., genes with high topological overlap) in an unsupervised fashion. Gene modules were visualized by heatmap plot (TOMplot) of the gene network topological overlap. Module relationships were summarized by a hierarchical clustering dendrogram and TOMplot of module eigengenes (MEs). Associations between gene modules and traits of interest were tested by correlating MEs to trait score. Module–trait correlations were visualized using a heatmap plot and only modules with trait relationship significance (R^2^) higher than 0.7 and a p-value ≤0.05 were considered for further analysis. Traits of interest used for WGCNA included: timeline from our previous study, previously reported CD14, CD86, CD206 and IL-10 expression measured by flow cytometry, and IL-10, IGF-1, MCP-1, IL-1β, TNF-α, PGE_2_ and SDF-1 concentrations quantified in conditioned SF and ISF (50). Module memberships (MM; correlation between each gene expression profile (GeTMM) and the ME of a given module as an indicator of the intramodular connectivity) and gene significance (GS; correlation between the gene expression profile (GeTMM) and the trait score (e.g. cytokine concentration in conditioned SF/ISF) as a measure of biological relevance) were calculated (58). Genes (network nodes) having MM ≥ 0.90, *P-*value *<* 0.05, and GS ≥ 0.5 were identified as intramodular hub genes (61). Gene ontology (GO) analysis was performed on the entire gene list derived from each module as described above using DAVID Bioinformatics Resources version 6.8 (62) to functionally annotate their biological processes (BP). Of note, no single time point was chosen to determine the module-trait correlations. The entire timeline of the study was itself a trait. Therefore, genes within each module were co-expressed at all time points and thus dominant (overrepresented) BPs for a given module were the same at all time points.

To predict upstream regulators relevant for each set of DEGs, analysis was performed using the Ingenuity Pathway Analysis software (IPA, 2018) (63). The analysis output provided a *P*-value of overlap, activation Z-scores, and the downstream targets for each predicted upstream regulator. Z-scores were used to predict activation state (activation or inhibition) of each upstream regulator/signaling pathway. Predicted upstream regulators were considered significant if they had *P* < 0.05 and activation Z-score >2 (activated) or <−2 (inhibited). Subsequently, we investigated overlap between the predicted upstream regulators for each set and the DEGs from the same set to identify potential regulators among those DEGs. Genes in common between the two analyses with Z-scores (generated by IPA) matching the direction of fold change (generated by DESeq2) were defined as potential regulators. To investigate the interaction and relationships between potential upstream regulators, all known protein–protein interactions were referenced and matched using STRING version 10.5 (64). Potential upstream regulators of high interaction were selected to have their protein expression assessed in synovium from inflamed joints treated with BMNC or DPBS, as a means of identifying candidate biomarkers of BMNC-mediated resolution. Synovial membrane samples were obtained from a related study in which BMNC therapy induced marked inflammation resolution (49) and represented inflamed joints treated with autologous BMNC or DPBS. IPA was also used to determine activated and inactivated signaling pathways, considering significance at *P* < 0.05 and activation Z-score >2 (activated) or <−2 (inhibited) and a -log(p-value > 1.3, which corresponds to p>0.05). For cases in which a large list of pathways met this criterion, those with a -log(p-value > 3 (FDR <0.01) were given priority attention.

### Immunohistochemistry

Formalin-fixed paraffin-embedded synovial membrane samples from inflamed joints of 6 horses treated with either BMNC or DPBS were sectioned at 5-7 µm and baked at 38°C for 48 hours. Sectioned tissues were processed with the BOND-MAX system (Leica Microsystems, Buffalo Groove, IL) using antibodies for the following gene-products, identified as key upstream regulators or key genes from most activated pathways: peroxisome proliferator-activated receptor γ (PPARγ; rabbit anti-human, clone 16643-1-AP, ThermoFisher Scientific), phospho- PPARγ (rabbit anti-human, clone PA536763, ThermoFisher Scientific), PPARγ co-activator 1 alpha (PPARGC1A; rabbit anti-human, clone PA5-38021; ThermoFisher Scientific), mevalonate kinase (MVK; rabbit anti-human, clone PA528650, ThermoFisher Scientific), 3-Hydroxy-3-Methylglutaryl-CoA Synthase 1 (HMGCS1; rabbit anti-human, clone PA529488, ThermoFisher Scientific), colony-stimulating factor 1 (CSF1; rabbit anti-mouse, clone PA5-95279; ThermoFisher Scientific), interleukin-1β (IL-1β; rabbit anti-human, clone P420B; Invitrogen), transcription factor MAFB (MAFB; rabbit anti-human, clone PA5-40756; ThermoFisher Scientific) and sirtuin 2 (SIRT2; rabbit anti-human, clone PA3-200; ThermoFisher Scientific). Positive controls included equine liver, heart, and kidney. Negative controls were prepared with mouse (for PPARγ, phospho- PPARγ, MVK, HMGCS1, PPARGC1A, IL-1β, MAFB, SIRT2 antibodies) or goat (CSF1) IgG (Santa Cruz Biotechnology, Inc.). Photographs of representative areas were scored by 3 experienced investigators for staining intensity (0-absent, 1-mild, 2-moderate, 3-intense) and distribution (0-absent, 1-scattered, 2-focal, 3- across the entire villi lining) as previously described (49). Composite scores for immunohistochemical data were presented as median and 95% confidence interval and analyzed by paired t-tests with significance set as *p* ≤ 0.05 using Prism GraphPad 7.

## Results

### Temporal Transcriptional Changes in BMNC Following Culture in SF and ISF

#### Differential Gene Expression

**Figure 2:**
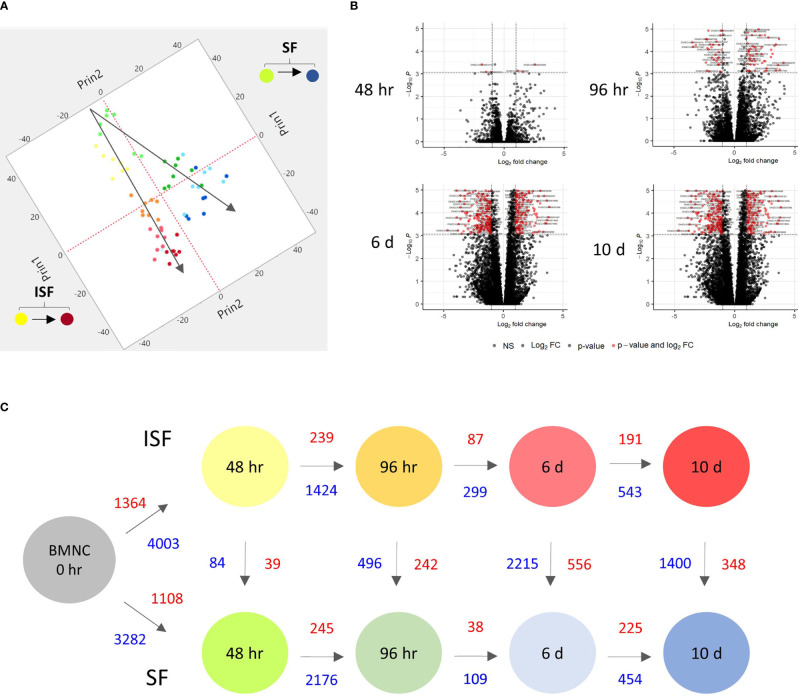
Kinetics of transcriptional signatures of BMNC cultured in SF and ISF. **(A)** Principal Component Analysis from 8456 genes differentially expressed (DEGs) by BMNC following culture in SF (green to blue dots; top) and ISF (yellow to red dots; bottom) for 10 days shows increasing divergence in the patterns of gene expression over time. Each dot color represents a different time point and each dot represents an individual horse. **(B)** Volcano plots depicting downregulated (left red quadrant) and upregulated (right red quadrant) DEGs in ISF compared to SF cultures at each time point, showing major changes at 6 days. **(C)** Schematic of changes in gene expression of BMNC cultured in SF (bottom) and ISF (top) for 10 days, depicting the number of DEGs over consecutive time points within groups (horizontal comparisons in SF or ISF) and between groups (vertical comparisons) at each time point (FDR ≤0.05). Numbers represent upregulated (red text) and downregulated genes (blue text) between the compared conditions.

**Figure 3:**
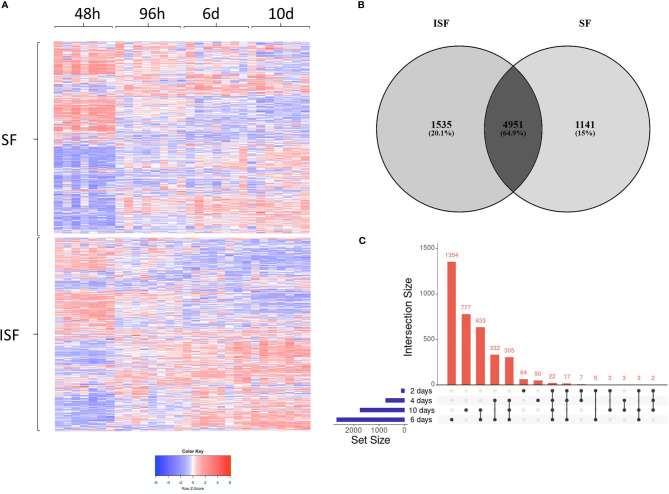
Differentially expressed genes (DEGs) in BMNC cultured in normal (SF) and inflamed autologous synovial fluid (ISF). **(A)** Heatmap of DEGs (FDR<0.05) identified among all possible comparisons (n=8456) between SF and ISF cultures over 10 days. The heatmap was created using Log10 transformed GeTMM values expressed on a color scale denoting high (red) and low (blue) expression. Each dataset (SF and ISF) included all DEGs displayed in a fixed position for comparison of the effect of culture medium over the same genes. **(B)** Venn diagram illustrating the intersection between DEGs identified by horizontal comparisons in either SF or ISF cultures. **(C)** Upset plots elucidating the intersection between DEGs identified by vertical comparisons. The nature of each intersection is indicated by the dots under the vertical bars, which denote the number of DEGs in each intersection, while horizontal bars represent the number of DEGs in each comparison.

Principal component analysis (PCA) of DEGs between BMNC cultured in SF and ISF showed clear differences in clustering patterns as early as 48 hours, and progressively diverged over time, representative of differences in BMNC response to normal and inflammatory environments (**Figure 2A**). Volcano plots depict up and downregulated DEGs when comparing ISF to SF cultures at each time point (**Figure 2B**). Vertical and horizontal comparisons were made, with the number of DEGs between any two conditions reported (**Figure 2C**). In vertical comparisons, ISF cultures were compared to their SF counterparts at each time point. Counts of upregulated genes were most remarkable at 96 hours and 6 days. In horizontal comparisons, each subset of BMNC was compared with its nearest time point to analyze gene expression variation from BMNC response along the timeline. From baseline to 48 hours, when myeloid progenitors in BMNC commit to the monocyte/macrophage lineage, the number of DEGs was highest among all time points, for both SF and ISF. The expression patterns of DEGs identified in all vertical and horizontal comparisons were also visualized by heat map (**Figure 3A**). Further, Venn diagrams were used to illustrate the intersection between DEGs identified by horizontal comparisons and revealed that SF and ISF cultures shared 64.9% of DEGs over the 10 days, while those expressed exclusively in the SF or ISF dataset represented 15.0% and 20.1%, respectively (**Figure 3B**). Upset plots elucidating the intersection between DEGs identified by vertical comparisons revealed that most DEGs were exclusively expressed in ISF cultures at 6 days (**Figure 3C**). An entire list of DEGs is available at **Supplementary Table 1**.

#### Co-Expression Network Analysis From BMNC in Response to Inflammation

**Figure 4:**
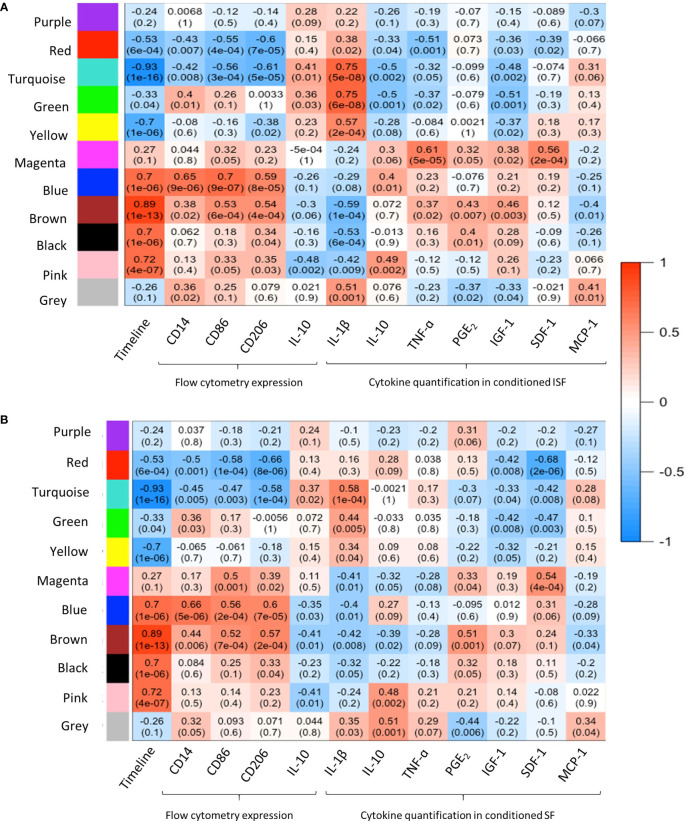
Weighted gene co-expression network analysis (WGCNA): module-trait relationships. **(A)** WGCNA of 18,038 genes in ISF identified 11 modules eigengene (ME), of which 6 were positively associated (R^2^≥0.7, p=≤005) with the assigned traits. The turquoise and green modules were positively associated with IL-1β quantification in ISF conditioned by BMNC. MEs blue, brown, black and pink were positively associated with the timeline. The blue module was also associated with CD86 expression assessed by flow cytometry (29). **(B)** In the SF dataset, analysis of the 16,318 genes identified the same 11 gene modules; however, timeline was the only trait with a positive association to MEs blue, brown, black and pink modules, as in ISF.

WGCNA provided further insights into the patterns of gene co-expression and the identification of genes with the highest interaction or connectivity (hub genes) among SF and ISF datasets separately (hub genes are denoted by bold cells in **Supplementary Table 2**). Co-expression analysis of 18,038 genes in ISF identified 11 module eigengenes (i.e., clusters) (**Figure 4A**). Among these, modules turquoise, green, blue, brown, black and pink were positively associated with three of the assigned traits. The turquoise and green modules were positively associated with IL-1β concentrations in conditioned ISF, and thus interpreted as having an overall pro-inflammatory nature. Modules blue, brown, black and pink were positively associated with the timeline. The blue module was also associated with CD86 expression assessed by flow cytometry, which denotes macrophage activation (50). In the SF dataset, analysis of the 16,318 genes identified the same 11 gene modules; however, the timeline was the only trait with a positive relationship to MEs, which like ISF included the blue, brown, black and pink modules (**Figure 4B**). Thus, the IL-1β-related green and turquoise modules are the standout, inflammation-associated differences between ISF and SF cultures, while the blue module also differed in the number of positively associated traits. Since events associated with inflammation resolution would only be present in an inflammatory environment, further dissection of WGCNA findings were centered on data from ISF cultures, while data from SF cultures were used as a point of comparison.

**Figure 5:**
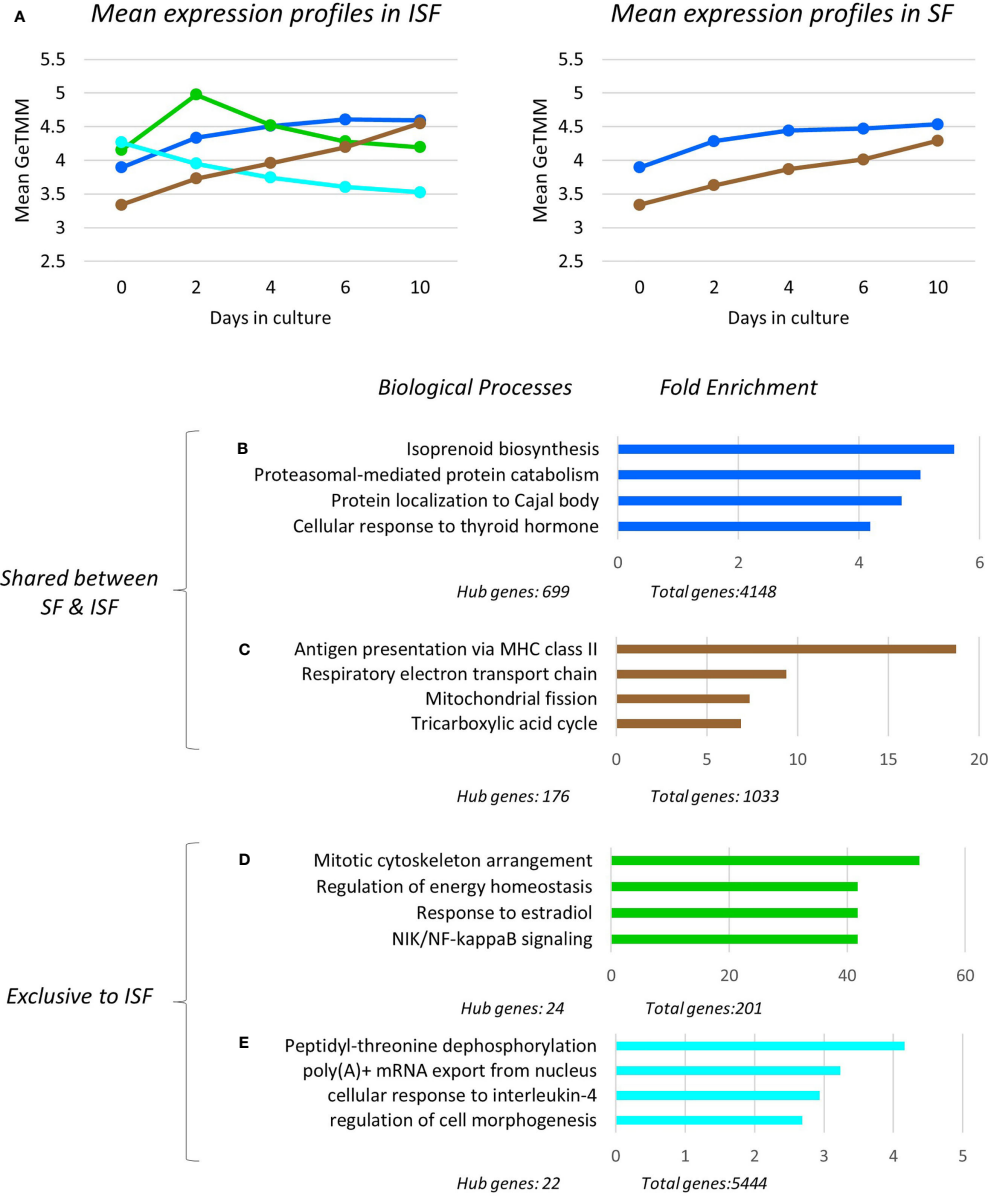
Expression profile, gene ontology enrichment and overrepresented biological processes within dominant modules. The green and turquoise modules were only significant in ISF and positively associated with IL-1β quantification in conditioned ISF, and overall associated with pro-inflammatory mechanisms. The blue and brown modules were significantly associated to the timeline for both SF and ISF, while the blue module was positively associated to CD86 expression in ISF. **(A)** Mean expression profiles of significant modules in SF and ISF derived from all transcripts in each cluster. In ISF, the presence of the pro-inflammatory green and turquoise modules are associated to increased mean expression of the homeostatic/pro-resolving blue and brown modules. Overrepresented Biological Processes (BPs) in the blue **(B)**, brown **(C)**, green **(D)**, turquoise **(E)** modules and their corresponding fold enrichment. The complete list of significant modules, BPs and related genes for each module is presented in **Supplementary Tables 2****,** **3**.

To assess the temporal behavior of each module, the mean expression profile (mean GeTMM values for all genes) for each module was plotted over time for both SF and ISF separately (**Figure 5A**). This comparison revealed that the blue and brown modules exhibited increasing mean expression profiles that similarly dominated over time in both SF and ISF, which however, were higher in ISF. The pink and black modules had lesser expression among modules identified which completely overlapped between SF and ISF, and therefore are not graphically represented. Additional comparisons for a given module between ISF and its SF counterpart also included the functional annotation of the genes within such modules (**Figures 5B–E**). The blue, brown, black and pink modules completely overlapped between ISF and SF regarding their gene list, HUB genes list (**Supplementary Table 2**) and functional annotations (**Supplementary Tables 3****,** **4**). Exclusive to ISF, the green module peaked at 48 hours, the same time at which the blue and brown modules started to exhibit increased expression in comparison to its SF counterpart. The mean expression profile in the ISF’s turquoise module progressively decreased from baseline. Functional annotation of genes within each module was then inspected (**Figures 5B–E**).

#### Modular Gene Ontology Enrichment and Overrepresented Biological Processes (BPs)

Overrepresented BPs were ranked based on fold enrichment and having an FDR <0.05 (**Supplementary Table 3**). For cases in which a large list of BPs met this criterion, BPs with FDR <0.01 were given priority attention. Fold enrichment was determined by comparing the background frequency of total genes annotated to a certain BP in the specified species to the sample frequency of genes under such BP. Overrepresentation was defined by a positive fold enrichment value (65). Since the gene list for modules blue, brown, pink and black completely overlapped between SF and ISF (**Supplementary Table 2**), overrepresented BPs in any of these modules were the same for both groups (**Figures 5B–E**). For the blue module, isoprenoid biosynthesis was the most overrepresented of the 79 BPs identified by GO. Given the high number of overall (n=4148) and hub genes (n=699) in this module, a diversity of BPs was identified within it, and is collectively discussed below. Of note, genes identified with pro-resolving functions in the related previous studies (*IL10* and *IGF1)* (49, 50) also allocated to the blue module. In the brown module, while the most overrepresented BP was “antigen presentation *via* MHC class II”, most BPs in this module related to mitochondrial response to oxidative stress and energy metabolism homeostasis. Overrepresented BPs in the pink and black modules constituted a minor list and were associated with a variety of cell homeostasis and housekeeping functions.

Exclusive to ISF cultures, BPs in the IL-1β-associated green module were primarily associated with macrophage response to damage, including mitosis, adjustment of lipid and glucose metabolism following circadian distress, activation of the amphireguling-STAT3 axis (GO:0032355~response to estradiol) and noncanonical NF-κB signaling, thus, likely a module with a pro-inflammatory signature. In the turquoise module, also associated with IL-1β production, overrepresented BPs reflected the response of myeloid progenitors to stress and IL-4 signaling, a key event in the response of pro-resolving macrophages, amplifying chromatin opening for enhanced mRNA transcription (44–46). In summary, ISF triggered an early pro-inflammatory response in BMNC progenitors (green module) leading to macrophage commitment and priming (turquoise module). These events enhanced the constitutive expression of homeostatic mechanisms from macrophages (blue and brown modules) required to counteract damage and recover homeostasis (**Figures 5A–E**).

#### Pathway Analysis, Upstream Regulators and Their Network Interactions

**Figure 6:**
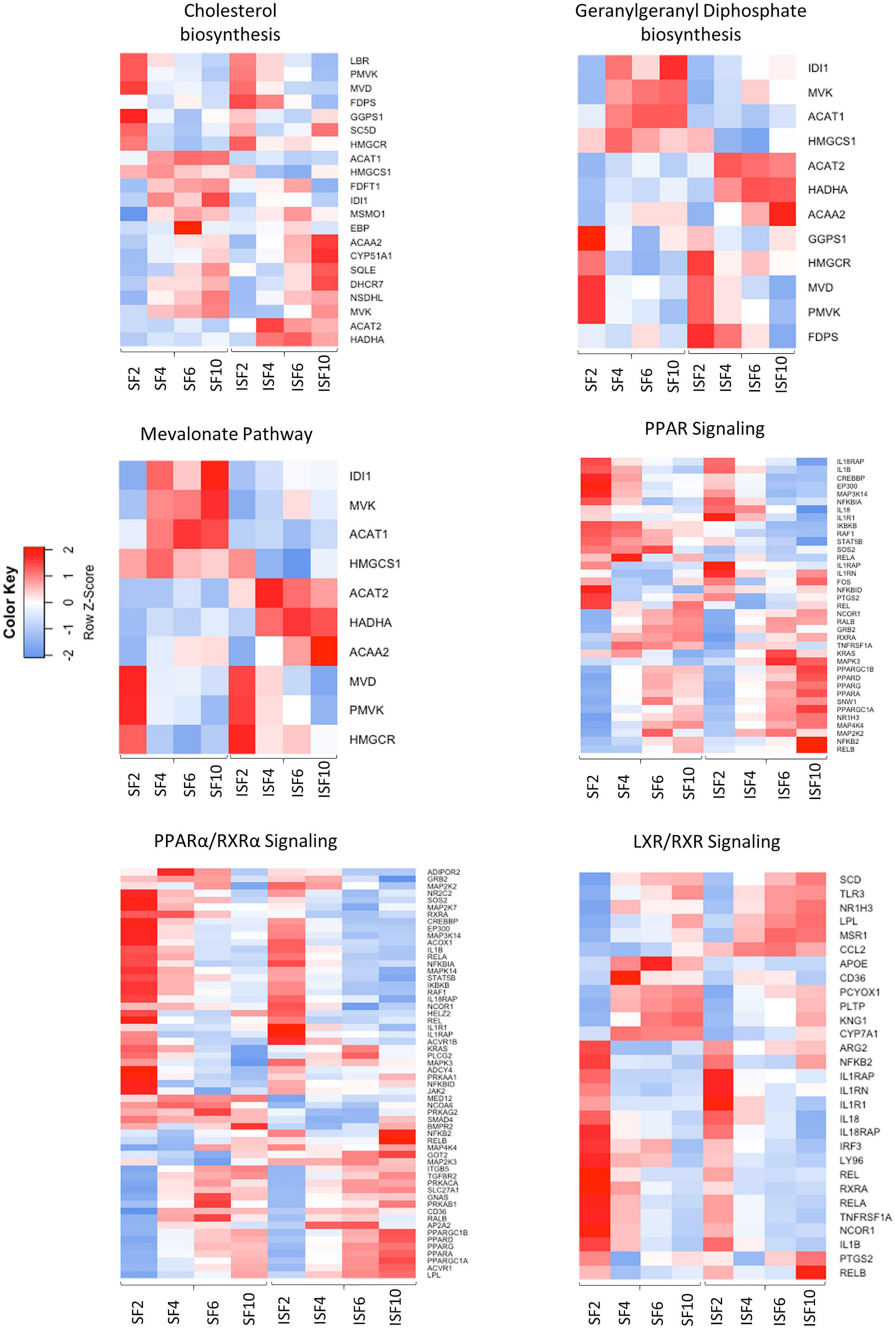
Heatmaps depicting the patterns of expression of genes involved in the most highly activated signaling pathways associated with resolution of synovitis, comparing BMNC cultured in SF and ISF. Further details are available at **Supplementary Table 4**.

Ingenuity Pathway Analysis revealed activated and inactivated pathways in SF and ISF cultures (**Table 1**, **Supplementary Table 4**). Our pathway analysis results from 0-48 hours (performed with IPA) agrees with findings from GO analysis and points repeatedly to activation of the mevalonate pathway and isoprenoid biosynthesis (superpathways of cholesterol biosynthesis, geranylgeranyl diphosphate biosynthesis, cholesterol biosynthesis I, II and III, and mevalonate pathway I). The patterns of expression of genes involved in these pathways, comparing BMNC cultured in SF and ISF (**Figure 6**), highlight the increased expression of genes such as *ACAA2, HADHA ACAT2* and *FDPS in ISF*, essential for the synthesis of isoprenoids and mitochondria beta-oxidation of fatty acids. Additional pathways activated at 0-48 hours included unfolded protein response in agreement with overrepresented BPs in the blue module, and estrogen biosynthesis, in agreement with the BP “response to estradiol” from the green module, peaking at 48 hours and progressively decreasing.

**Table 1:** Top 3 most activated or inactivated pathways (Z-score *>* 2.0 or *<* -2.0; -log (p-value) > 1.3 = p>0.05) identified by IPA from DEGs between consecutive timepoints in SF and ISF.

*Time*	ISF		SF	
	*Activated * *z-score; -log (p-value)*	*Inhibited * *z-score; -log (p-value)*	*Activated * *z-score; -log (p-value)*	*Inhibited * *z-score; -log (p-value)*
***0-48 hr***	Superpathway of cholesterol biosynthesis (4.14; 10.3)	Signaling by Rho GTPases(-3.35; 8.00)	Superpathway of cholesterol biosynthesis (4.24; 8.74)	IL-5 production (-3.00; 4.43)
	Geranylgeranyl diphosphate Biosynthesis I (via Mevalonate) (3.31; 4.88)	Leukocyte extravasation signaling (-3.20; 14.9)	Geranylgeranyl diphosphate Biosynthesis I (via Mevalonate) (3.31; 5.83)	Apoptosis signaling (-2.55; 4.13)
	Mevalonate Pathway I (3.00; 4.54)	IL-5 production (-3.317; 5.43)	Mevalonate Pathway I (3.00; 5.34)	Signaling by Rho GTPases (-2.35; 4.12)
***48-96 hr***	PPAR signaling (3.41; 9.22)	Acute Phase Response signaling (-4.42; 5.96)	LRX/RXR activation (3.71; 4.08)	Natural Killer cell signaling (-5.85; 4.12)
	PPARα/RXRα activation (3.08; 9.22)	Natural Killer Cell signaling (-4.13; 6.23)	PPAR signaling (3.41; 9.89)	IL-6 signaling (-5.24; 14.9)
	LXR/RXR activation (2.67; 3.27)	IL-6 signaling (-4.01; 10.50)	PPARα/RXRα activation (2.59; 12.00)	Acute Phase Response signaling (-4.87; 7.31)
***96 hr- *** ***6 d***	Heparan Sulfate Biosynthesis (Late Stages) (2.0; 1.79)	IL-6 signaling (-2.23; 2.08)	-----------------	-----------------
	Heparan Sulfate Biosynthesis (2.0; 1.66)	PRRs in Pathogen Recognition (-2.0; 1.69)	-----------------	-----------------
***6-10 d***	Cell Cycle: G2/M DNA Damage Checkpoint Regulation (2.33; 5.27)	Cell cycle control of chromosomal replication (-3.60; 8.78)	GP6 Signaling Pathway (3.05; 4.83)	EIF2 signaling (-5.456; 41.90)
	Unfolded Protein Response (2.44; 3.23)	Kinetochore Mataphase signaling (-2.98; 16.61)	Osteoarthritis Pathway (2.53; 4.15)	Oxidative Phosphorylation (-3.00; 2.81)
	Netrin signaling (2.23; 1.39)	Mitotic Roles of Polo-Like Kinases (-2.12; 6.89)	Tumor Microenvironment Pathway (3.31; 2.80)	Autophagy (-2.23; 0.00)

In agreement with GO, dominant responses related to inflammation-derived oxidative stress and an antioxidant response relying on lipid biosynthesis and activation of the PPAR signaling pathway. While the IL-6 pathway inhibition was common to both SF and ISF, it was a more frequent in ISF cultures. A detailed list of activated and inactivated pathways is presented on **Supplementary Table 4**.

From 48-96 hours, the PPAR- signaling pathways were repeatedly identified, which agrees with the identification of *PPARG* as highly connected upstream regulator, depicting the activation of the PPAR-γ and PPAR-α signaling pathways. Comparisons for BMNC cultured in SF and ISF for the expression of genes involved in these pathways (**Figure 6**), highlight the higher expression of PPAR genes in ISF. It also highlights the higher expression of *NFKB2* and *RELB* genes, encoding for drivers of non-canonical NF-κB signaling, which has essential pro-resolving functions. After 96 hours of culture in ISF, a mix of pathways involved in cartilage metabolism (Heparan Sulfate Biosynthesis), cellular homeostasis/inflammation resolution (Cell Cycle: G2/M DNA Damage Checkpoint Regulation, Unfolded Protein Response) and leukocyte migration during inflammation (Netrin Signaling) were observed to be activated. This mixed profile may have resulted from continuously challenging BMNC with ISF every 48 hours as performed in our model (50). Inhibited pathways included leukocyte extravasation signaling, IL-6 across the time course, IL-15 production and acute phase response signaling, key players in the development and maintenance of synovitis and degenerative processes observed in osteoarthritis. While the “Osteoarthritis Pathway” was identified as the third most activated pathway in SF at 6-10 days, neither the model used in our study, nor the list of genes involved in such functional annotation support such a finding.

**Figure 7:**
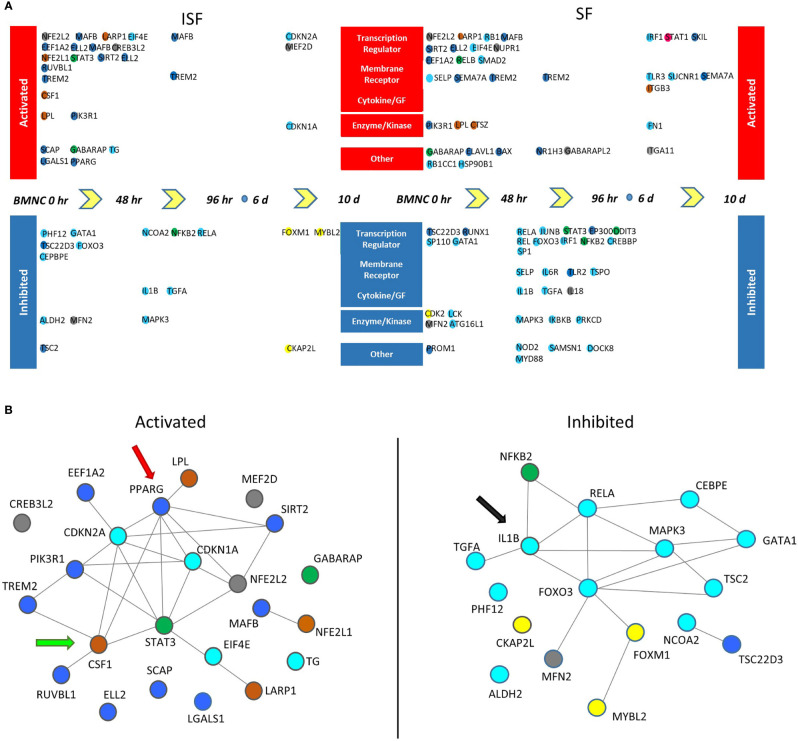
Potential upstream regulators of genes differentially expressed between consecutive time points (DEGcts) in SF and ISF cultures. **(A)** Time window specific identification of upstream regulators analysis of the DEGcts using Ingenuity Pathway Analysis (IPA). The colored circles are upstream regulators identified for each comparison of consecutive time points with an activation or inhibition Z-score (*>* 2.0 or *<* -2.0), respectively (**Supplementary Table 5**). Colors of the circles correspond to colors of the module eigengene from WGCNA to which that specific gene associated. Activated upstream regulators are shown at the top and those inhibited at the bottom. Upstream regulators were grouped into five categories: `Transcription regulator’, `Membrane receptor’, `Cytokine/Growth Factor (GF)’, ‘Enzyme/Kinase’ and `Other’. **(B)** Interaction network among activated (left) and inhibited (right) upstream regulators in BMNC cultured in ISF. Color coding of nodes relate to the corresponding module eigengene. Interaction networks reflect a response to inflammation-induced oxidative stress associated with proliferation and differentiation of BMNC into macrophages (CSF1 - green arrow), and a PPARγ-reliant inflammation resolution (red arrow), associated with inhibition of the IL-1β signaling pathway (black arrow) as suggested by complimentary analyses.

Twenty-three potential upstream regulators were identified as activated (p <0.05 and a Z-score ≥2) in ISF and 35 in SF (**Figure 7A**, **Supplementary Table 5**, **Supplementary Figure 1**). Within these, 5 transcription factors (*EIF4E, LARP1*, *MAFB, NFE2L2* and *SIRT2* genes), the transmembrane receptor *TREM2*, the enzymes *LPL* and *PIK3R1*, and the multifunctional receptor *GABARAP* were conserved between ISF and SF. Inactivated upstream regulators (p <0.05 and a Z-score ≤ 2) were also identified in both ISF (n=17) and SF (n=32). Within these, *IL1B* and its downstream signaling transcription factor *RELA*, the mitochondria fission receptor *MFN2*, and the transcription factor *GATA1* were conserved between ISF and SF.

Interaction networks generated by STRING among activated and inhibited upstream regulators in ISF cultures revealed predicted interactions between upstream regulators in and outside their same module (**Figure 7B**). Among activated upstream regulators, the blue module was overrepresented in ISF (50%; including *PPARG*, *SCAP, MAFB* and *SIRT2*) followed by the brown module (*CSF1, LARP1, LPL, NFE2L1*) and turquoise module (including *CDKN1A, CDKN2A, EIF4E, TG*) equally representing 16.6% of upstream regulators, while the grey (12.5%; *CREB3L2, MEF2D, NFE2L2*) and green (8.3%; *STAT3* and *GABARAP*) modules were minimally represented. Amongst inhibited upstream regulators in ISF (**Figure 7B**), the turquoise module was overrepresented (70.5%) and largely related to the IL-1β/NF-κB signaling pathway. Overall, such interactions underscore the proliferation and differentiation (*CSF1*) of BMNC into macrophages (50), and their response to inflammation-induced oxidative stress (*PPARG, NFE2L1*) *(*43, 66, 67) involving changes in lipid metabolism (*LPL*) (68). Such a response likely mediates resolution, at least partially in a PPAR-γ-reliant manner, downregulating the IL-1β/NF-κB signaling pathway (*IL1B* and *RELA* genes) (43).

### Tissue Expression of Upstream Regulators and Genes Central to Activated Pathways

**Figure 8:**
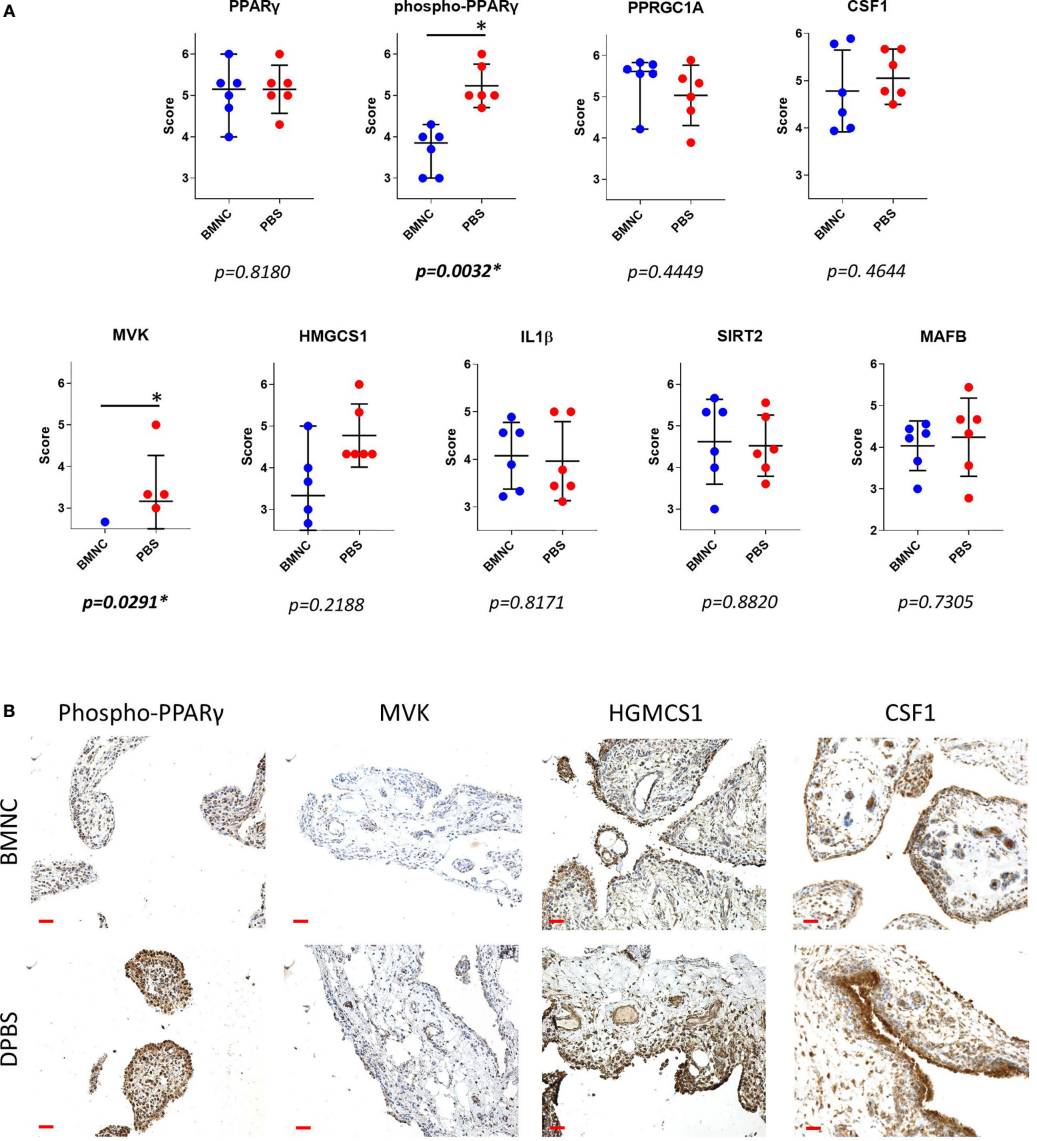
Immunohistochemistry of synovial membranes from 6 horses with experimental synovitis treated with BMNC or DPBS. Marked resolution of inflammation was evident following BMNC therapy (31). Selected targets were potential upstream regulators of high network interaction that were differentially expressed in ISF cultures over consecutive time points, or central drivers of most activated pathways. **(A)** Scatterplots of composite staining scores (median, 95% confidence interval) for PPARγ (*PPARG gene*), phosphorylated PPARγ, PPARγ co-activator 1 alpha (*PPARGC1A gene*), colony stimulating factor 1 (*CSF1 gene*), mevalonate kinase (*MVK gene*), 3-Hydroxy-3-Methylglutaryl-Coenzyme A Synthase 1 (*HMGCS1 gene*), interleukin-1β (*IL-1β gene*), sirtuin 2 (*SIRT2 gene*), and transcription factor MAFB (*MAFB gene*). Each dot in the scatterplot represents the composite score for each individual horse. Tissue expression for phosphorylated PPARγ (*p=0.0032*) and mevalonate kinase (*p=0.0291*) were lower in BMNC-treated joints. **(B)** Representative sections of synovium from inflamed joints treated with BMNC or DPBS and stained for selected markers detailed above (scale bars 100µm).

From the patterns of gene expression for each activated upstream regulator assessed over time (GeTMM counts and fold change, **Supplementary Table 6**), those with a high network interaction and exhibiting evident fold changes over time (decreasing or increasing) were selected for immunohistochemistry. Genes identified as essential drivers of the most activated pathways, were also selected as IHC targets. Ultimately, available immunohistochemistry targets with antibodies known to cross-react with equine proteins included PPARγ, phospho-PPARγ, PPARGC1A, MVK, HMGCS1, IL-1β, MAFB, SIRT2, and CSF1. Tissue expression for phosphorylated PPARγ (*p=0.0032*) and mevalonate kinase (*p=0.0291*) were lower in BMNC-treated joints (**Figure 8**), likely because the resolution process had already been achieved, and is similar to the patterns of expression of IL-10 in our previous study using the same synovial samples (49). None of the remaining histochemical findings reached statistical significance. Tissue expression for PPARGC1A was consistently higher in BMNC-treated joints for 5/6 horses compared to those treated with DPBS. In contrast, CSF1 and MAFB tended toward higher expression in DPBS-treated joints. No differences were observed in the staining patterns for SIRT2 and IL-1β.

## Discussion

In this study, we identified differential transcriptional signatures of BMNC in response to ISF and SF. These same BMNC were previously shown to resolve synovitis following exposure to an inflamed synovial environment *in vivo* and *in vitro* (49, 50). We demonstrate a temporal behavior of co-expressed gene networks and their association with traits from our previous studies (49, 50), as well as with the expression of key proteins in the synovium by immunohistochemistry. Our findings illustrate the elaborate balance of pro- and anti-inflammatory mechanisms shifting dominance through the recovery of joint homeostasis. BMNC responded to ISF with an early pro-inflammatory response (green module), characterized by a short spike in the expression of NF-ƙB-related genes, coincident with the peak of IL-1β secretion in conditioned ISF (50). This response was associated with increased expression of the blue and brown modules, 2 gene networks with homeostatic functions comprising known drivers of resolution, which were more highly expressed in ISF and were dominant among activated upstream regulators. Significant differences in the expression of phosphorylated PPARγ and mevalonate kinase in synovial membranes from inflamed joints treated with BMNC, and equivalent IL-1β staining between BMNC- and DPBS-treated joints, emphasize the fine tuning of so-called pro-inflammatory pathways that must remain active at physiological levels during the resolution process. These observations highlight the differences between the pro-resolving effects associated with BMNC therapy compared to the anti-inflammatory effects observed following clinical treatment with corticosteroids (30, 49–51, 69, 70).

The short spike of expression of the pro-inflammatory green module observed in ISF, is essential to trigger a cascade of events that culminates in a pro-resolving response. NF-ƙB-related and mitogen genes co-expressed in the green module (**Supplementary Table 3**) play a key role in promoting the proliferation of macrophages necessary to counteract damage (24, 25). NF-ƙB-related genes also induce increased expression of genes with anabolic and anti-inflammatory functions within the blue and brown modules, which have critical roles in driving resolution of joint inflammation. The top four overrepresented BPs in the green module relate to a group of genes sharing important mitogenic activity. Genes such as *MAD1L1* and *CHAMP1* encode proteins that interact and regulate cell structure organization preceding mitosis (71, 72). *NR4A3* and *NR1D2* encode transcriptional activators involved in proliferation, survival and differentiation of myeloid progenitor cells, and in adjusting myeloid progenitor cell metabolism to oxidative stress (73, 74). NR1D2 does so by activation of IL-6 transcription, which is also required to induce expression of the IL-4 receptor and related downstream regulatory functions of macrophages, including their self-renewal (75–77). The BP “response to estradiol” was characterized by the expression of the amphiregulin (*AREG*) and *STAT3* genes. Macrophages are an important source of amphiregulin produced during acute inflammation. The AREG/ERK/STAT3 signaling axis is required for the differentiation of progenitor cells during tissue repair and establishing a pro-resolving response (78–81). *AREG* was more highly expressed in ISF and exhibited progressively decreasing expression over time, as the resolving response progressed (**Supplementary Table 6**). Additionally, estrogen accelerates the resolution of inflammation through the regulation of IL-10/STAT3-mediated deactivation of pro-inflammatory responses, such that post-menopausal women are prone to developing chronic inflammation (82). STAT3 was the activated upstream regulator in ISF cultures with the highest connectivity. The NIK/NF-ƙB signaling in the green module was highlighted by the expression of *RELB* and *NFKB2*. Both RELB and NFKB2 are subunits of the non-canonical NF-κB signaling, which in macrophages, can exert both pro- and anti-inflammatory effects (83, 84). Non-canonical NF-κB signaling is critical to produce SDF-1α and recruit monocytes to the site of damage immediately following injury (83). Further, during monocyte-macrophage differentiation, non-canonical NF-κB signaling prevents hyperactivation of new macrophages by accelerating the removal of RelA and c-Rel (canonical NF-κB subunits) from pro-inflammatory gene promotors preventing overt inflammation. As such, blocking non-canonical NF-κB by inactivation of its IKKα subunit results in increased inflammation (84). In both SF and ISF cultures, *RELB* expression was positively regulated, with decreasing expression over time, while *RELA* was downregulated (**Figure 6**; **Supplementary Table 6**). Combined, these signatures illustrate a fraction of molecular drivers of the acute response of BMNC to inflammation, which also sets the stage for establishing a pro-resolving response.

Increased expression of the blue module in response to inflammation, in parallel with the surge of the green module, and over a timeline associated with resolution in our previous studies (49, 50), suggests a pro-resolving identity. Genes encoding established drivers of joint inflammation resolution (IL-10, IGF-1) allocated to the blue module (**Supplementary Table 2**). A major functional signature of this module was the activation of the mevalonate pathway and isoprenoids biosynthesis, comprised by the expression of genes encoding central drivers of the mevalonate/isoprenoid pathway (*COQ2, HMGCR, FDPS, HMGCS1, GGPS1, MVK, PDSS1, PDSS2, GGDPS1, FDPS, ACAA2, HADHA* and *ACAT2*. In agreement, the super pathway of cholesterol biosynthesis, geranylgeranyl biosynthesis (an isoprenoid) and mevalonate pathway had the highest activation scores in ISF cultures between 0 and 48 hours, as detected by IPA. The roles of the mevalonate pathway in steroidogenesis, counteracting oxidative stress and inflammation resolution, are well documented in the macrophage response to damage and inflammation resolution (85–88). Deficiency of mevalonate kinase (MVK), a key enzyme in the mevalonate pathway, causes reduced synthesis of isoprenoids, leading to mitochondrial damage, subsequent oxidative stress and severe inflammation (89, 90). Importantly, exogenous isoprenoid treatment in models of inflammation induces decreased oxidative stress and production of inflammatory markers, by increasing expression of the NF-κB inhibitor IκBα and antioxidant selenoproteins (86, 91–93). Gene expression for MVK and HMGCS1, key enzymes in the mevalonate pathway, exhibited a similar expression profile between themselves (**Figure 6**; **Supplementary Table 6**). These enzyme genes were more highly expressed in SF, suggesting that inflammation negatively affects this pathway. A similar pattern of gene expression for *IL10* and *IGF1* was observed in our previous study, in which both genes were more highly expressed in SF than ISF (50). However, concentrations of IL-10 and IGF-1 in ISF were higher than in SF, because the decreased relative production in ISF was compensated for by higher macrophage counts (50). Our ongoing lipidomic study on BMNC-conditioned SF and ISF from the same samples used in this study may help elucidate if a similar context applies for isoprenoid production. Interestingly, it was recently evidenced that transcriptional dysregulation of the mevalonate pathway is a key signature of the overt inflammation caused by SARS-CoV-2 infection, highlighting its importance for inflammation resolution (88). Differences in MVK expression detected in synovial membranes suggests that the *in situ* activity of MVK in synovitis resolution happens earlier in time, as suggested by our pathway analysis and *in vivo* study (49).

The second overrepresented BP in the blue module “proteasome ubiquitin-dependent protein catabolism” was comprised of genes that characterize the formation of the 26S proteasome. Inflammation-derived oxidative stress damages nascent proteins that become misfolded and targeted for degradation (94). The 26S proteasome is essential for the degradation of these proteins, preventing aggregate formation, which is part of the pathogenesis of several conditions (94). Such observation from GO analysis agrees with the IPA findings where the “Unfolded Protein Response” was identified among the most activated pathways following isoprenoid biosynthesis through the mevalonate pathway. The next overrepresented BP was “protein localization to the Cajal body”. These are coiled bodies found in the nucleus of proliferating or metabolically active cells and are implicated in telomere homeostasis (95, 96). This BP was represented primarily by genes encoding chaperonin containing tailless (CCT) proteins that are critical regulators of telomerase folding and trafficking (97). Depletion of CCT proteins cause Cajal body and telomerase mislocalization and failure of telomere elongation (97). CCTs are also required for folding of cytoskeletal proteins during cell proliferation (98). The fourth dominant BP, “response to thyroid hormone stimulus”, reflects the effects of triiodothyronine on regulation of macrophage maturation and responses. Such responses include controlling cell migration and conferring protection against endotoxemia and LPS exposure, in great part through proliferation of tissue resident macrophages (99, 100). In addition, half of the genes characterizing this BP encode for cathepsins that mediate endolysosomal protein degradation. In summary, the dissection of a minute part of the blue module, illustrates the effect of isoprenoids and thyroid hormones in improving metabolism and performance of BMNC-derived macrophages to counteract the effects of inflammatory oxidative stress (85, 89, 90, 99, 100). This response is, at least partially, achieved by adjusting proteostasis through the 26S proteasome and Cajal bodies, preventing degenerative protein aggregate formation during increased cell transcription, proliferation and metabolism in response to inflammation (94, 97, 98).

The signature of the brown module were BPs that comprised of a series of gene groups encoding proteins that regulate the mitochondrial respiratory chain and mitochondria-mediated regulation of energy metabolism (101, 102). There is growing evidence of the pivotal role of mitochondria in energy metabolism adjustments required for inflammation resolution as shown in the brown module (103–105). In the face of inflammatory challenges, enhanced cell respiration induces oxidative stress, activating alternative sources of energy driving gluconeogenesis and enhancing mitochondrial fatty acid oxidation (101, 102) as denoted by the increased expression of genes such *ACAA2* and *HADHA* in ISF cultures (**Figure 6**). These genes were, however, associated with the isoprenoid biosynthetic pathway, highlighting the recently reported role of mitochondrial isoprenoid biosynthetic process (106). Enhancing the mitochondrial respiratory chain is a homeostatic mechanism that prevents mitochondrial DNA damage and its subsequent cytosolic and extracellular release signaling through damage-associated molecular pattern (DAMP) receptors (107). The peroxisome proliferator-activated receptor-gamma (PPAR-γ) co-activator 1-α (PPRGC1A; *PPRGC1A* gene), a master regulator of mitochondria biogenesis and liver gluconeogenesis was among the outstanding genes involved in mitochondrial regulatory functions listed in the brown module (101) and exhibited a trend for higher expression in BMNC treated joints (**Figure 8**).

Following interaction with PPRGC1A, PPAR-γ exhibits increased activity, interacting with a multitude of transcription factors and PPAR-γ responsive elements (43). PPAR-signaling was an important activated pathway identified by IPA in both SF and ISF cultures between 48 and 96 hours (**Table 1**, **Figure 6**), denoting its homeostatic functions. Among different PPARs, PPAR-γ was one of the most highly connected upstream regulators. In macrophages and other cells, PPAR-γ signaling is a cornerstone of tissue repair and inflammation resolution, exhibiting myriad functions that are either PPAR-γ-mediated or -dependent (43). Examples include shifting the production of pro-inflammatory cytokines towards anti-inflammatory and pro-resolving mediators, driving apoptosis and clearance of neutrophils, enhancing macrophage traffic, recruitment, phagocytosis and efferocytosis, improving mitochondrial respiratory performance, and the overall transcriptome of a regulatory response driving recovery of homeostasis (43, 47, 101, 108). Of note, some isoprenoids, the signature of the blue module, as well as some specialized pro-resolving molecules, signal through PPAR-γ, conferring increased production of IL-10, resistance to inflammatory stimuli and attenuated NF-ƙB activation following LPS stimulation (43, 81, 109–111). Gene expression for *PPARG* was higher in ISF compared to SF. Significantly lower staining for phospho-PPAR-γ expression in synovial membranes detected by IHC, combined with the timing at which *PPARG* was identified as an Upstream Regulator, suggest that its activity modeling synovitis resolution almost overlap with the acute phase of inflammation. Importantly, PPAR-signaling findings from IPA at 48-96 coincide with the time at which pro-resolving effects were observed in our previous *in vivo* and *in vitro* studies (49, 50). Histochemical findings for phospho-PPAR-γ and PPRGC1A in BMNC compared to DPBS-treated controls suggests PPAR-γ signaling was not only a BMNC response to the inflamed synovial environment, but also part of the beneficial effects of BMNC on treated joints. Moreover, there is recent evidence that *PPRGC1A* expression is required for chondrocyte metabolism and cartilage homeostasis, with *PPRGC1A* knockouts exhibiting delayed endochondral ossification, disruption of physeal morphology and severe premature osteoarthritis (112).

Also in the brown module, *CSF1* was identified as an upstream regulator gene and was more highly expressed in ISF than SF cultures. The marked proliferation of BMNC in ISF observed *in vitro* (50) may also be a response from CSF-1 signaling (50). Following a spike of proliferation, stimulation of macrophage progenitors with interferon-gamma (IFN-γ) or LPS induce maturation and cell cycle arrest, increasing MHC-II expression and developing the capability to quickly respond to inflammatory stimuli and antigen presentation (113). Given that synovitis in our model was induced by injection of LPS, it is not surprising that “antigen processing and presentation of polysaccharide antigen *via* MHC class II” was a dominant BP in the brown module, particularly considering that IFN-γ was not detected by immunoassay in conditioned SF or ISF in a preliminary screening performed in our study (50). Combined, these observations from the brown module highlight the importance of macrophage proliferation and maturation, and PPRGC1A/PPAR-γ signaling during macrophage-mediated joint homeostasis, identifying the need for further investigation of the therapeutic roles of PPAR-γ-agonists in the recovery of joint health.

The combined overrepresented BPs in the turquoise module, exclusive to ISF, reflect the dynamic of serine/threonine phosphorylation, autophosphorylation and dephosphorylation during cytokine signaling and subsequent mRNA transduction (114). In our study, these events happened in response to IL-4 (50). IL-4 signaling regulates the establishment of a pro-resolving response in macrophages by adjusting chromatin conformation and access for RNA transcription initiation (45). It also represses the expression of classical pro-inflammatory genes, decreasing inflammasome activation, IL-1β production and pyroptosis (44). Alternatively, IL-4 signaling enhances DNA binding of RNAPII-pS2 and RNAPII-pS5, and H3K27Ac as a positive epigenetic modification that leads to increased expression of several gene networks with a pro-resolving function (44). STAT6, which also allocates to the turquoise module, is a major regulator of IL-4 (likely through IL-4r expression) and PPAR-γ signaling (46). STAT6 facilitates binding of PPAR-γ to DNA, which, like IL-4, increases the number of regulated genes and the magnitude of response from macrophages (44, 46, 47, 115). IL-4r expression was increased in BMNC cultured in both SF and ISF, but was significantly higher in ISF (50), in agreement with the overall findings in this module. We did not, however, detect gene expression for IL-4 or the IL-4r agonist IL-13. Also, neither IL-4 nor IL-13 were detected in the synovial fluid (SF or ISF) in our *in vitro* and *in vivo* studies (29,31). Collectively, these findings suggest that, if produced by cells in the joint other than BMNC, their concentrations were either below detectable limits or absent, raising the possibility of signaling by an unidentified IL4r agonist or an unconsidered pathway with equivalent gene ontology. The decreasing mean expression of the turquoise module in relationship to the increasing mean expression of the blue and brown modules, indicates that the biological processes included in the turquoise module are primarily required for priming the macrophages in BMNC towards a pro-resolving response (45).

*IL1B* gene expression in BMNC and protein expression in synovial fluid coincided in time and duration, peaking at 24 hours and progressively decreasing over time (49, 50). The *IL1B* gene was identified as a centerpiece among inhibited upstream regulators in ISF. Interestingly, IL-1β expression in the synovial membrane at 6 days following LPS model induction remained evident in both BMNC- and DPBS-treated joints. This finding suggests that the physiological levels of IL-1β expression required for homeostasis were conserved and not blocked, as commonly observed with the use of corticosteroids (51). Staining patterns for the other selected markers were inconclusive due to variability between individual horses. Although our experimental design was aimed at minimizing variability by including only horses of the same breed and a narrow range of age, variability is expected when working with human populations and animal models. Inbred mice poorly mimic the inflammatory reaction of people (116). While this study comprised a small cohort, the horse is an excellent model for the translation of inflammation (117) and an established model for the study of degenerative and inflammatory joint disease (1, 118). In agreement with our *in vivo* (49) and *in vitro* (50) findings using the same horses, the combined effects of gene networks comprised by each module depict a pro-resolving response, dissecting some important signatures of this process, and identifying candidate biomarkers of synovitis resolution, such as PPAR-γ and MVK expression and synovial fluid quantification of isoprenoids. The overall agreement between different analytical tools used in this study (between gene ontology of WGCNA-derived modules with IPA pathways and upstream regulator analysis) reinforce the significance of our data. However, future mechanistic studies are necessary to further determine the specific roles of pathways and biological processes identified in our study. A study of the transcriptome analysis from synovial samples from naturally inflamed joints treated with BMNC and DPBS would complement our current observations and further illustrate the molecular drivers of the synovial response to BMNC injection.

## Conclusion

Our current data suggest that BMNC-derived mechanisms of resolution are primarily represented by constitutively expressed homeostatic mechanisms, whose expression is enhanced to counteract tissue damage. These homeostatic mechanisms translate into macrophage proliferation, enlarging the “macrophage army” to fight aggressors, also improving their general and mitochondrial metabolism to better resist the challenges of inflammatory oxidative stress. Such effect is partially achieved through the synthesis and signaling of lipid mediators that promote recovery of homeostasis. Further exploration of BPs and pathways not dissected in this study may identify additional targets for future investigations. The combined findings of our equine studies (27, 49, 50) and human clinical trials (48, 119) highlight the long-lasting and superior pro-resolving effects of BMNC in the treatment of arthritic conditions. This study reveals important transcriptional signatures of BMNC-induced resolution of synovitis and reinforce that pro-resolving macrophages do not fit within commonly described pro- or anti-inflammatory phenotypes established in artificial *in-vitro* systems (120, 121). Current knowledge, including our study, suggests that *in vivo*, macrophages are by default homeostatic cells that, following injury, drive inflammation with the purpose of counteracting tissue aggressors, further guiding inflammation resolution and recovery of homeostasis (24, 25, 39, 122–125). Our study also highlights candidate mechanisms by which BMNC provide lasting improvement in patients with OA. Therapeutic enhancement of PPAR-γ signaling in joints with chronic inflammation may represent a novel strategy for resolving joint inflammation. Defining multiple mechanisms of macrophage-mediated synovitis resolution may provide means to develop pharmacological pro-resolving therapies, bypassing the need for more invasive bone marrow aspiration and further advancing the treatment of many inflammatory arthropathies, not just OA.

## Data Availability Statement

The datasets generated for this study are available on request to the corresponding author. The current RNAseq data was deposited in the Gene Expression Omnibus (GEO; GSE18552) repository.

## Ethics Statement

This study was conducted in compliance with the Animal Welfare Act and the approval of the Virginia Tech Institutional Animal Care and Use Committee.

## Author Contributions

BCM, JNM, and LAD designed studies. BCM obtained and processed the samples. TSK and SCL mapped and quantified the sequenced libraries. HE-SA and SCL performed DEGs analysis. BCM and HE-SA performed functional genomics, figures preparation, data interpretation and conceptualization. KES performed immunohistochemical assays, which were scored by BCM, HE-SA, and KES. BCM prepared the manuscript. All authors edited, reviewed, and approved the manuscript.

## Funding

This study was supported by the Grayson-Jockey Club Research Foundation and the Virginia-Maryland College of Veterinary Medicine Internal Research Competition. BM received graduate assistantship support from the Interdisciplinary Graduate Education Program at Virginia Tech, the Virginia-Maryland College of Veterinary Medicine and the College of Agriculture, Food and Environment at the University of Kentucky.

## Conflict of Interest

The authors declare that the research was conducted in the absence of any commercial or financial relationships that could be construed as a potential conflict of interest.

## Publisher’s Note

All claims expressed in this article are solely those of the authors and do not necessarily represent those of their affiliated organizations, or those of the publisher, the editors and the reviewers. Any product that may be evaluated in this article, or claim that may be made by its manufacturer, is not guaranteed or endorsed by the publisher.

## Abbreviations

OA, osteoarthritis; BMNC, bone marrow mononuclear cells; IL, interleukin; IGF-1, insulin-like growth factor 1; PGE_2_, prostaglandin E_2_; NF-ƙB, nuclear factor – kappa B; SF, normal synovial fluid; ISF, inflamed synovial fluid; DPBS, Dulbecco’s phosphate buffered saline; LPS, lipopolysaccharide; CSF1, colony-stimulating factor 1; SDF-1, stromal derived factor-1; IL-1ra, interleukin-1 receptor antagonist; WGCNA, weighted gene co-relation analysis; FPKM, fragments per kilobase of transcript per million mapped reads; DEGs, differentially expressed genes; GO, gene ontology; IPA, ingenuity pathway analysis; PPAR-γ peroxisome proliferator-activated receptor-gamma; PPARGC1A, PPAR-γ co-activator 1-α; TLR3, toll-like receptor 3; MAFB, transcription factor MAFB; SIRT2, sirtuin 2; FN1, fibronectin 1; PCA, principal component analysis; BP, biological process; STAT, signal transducer and activator of transcription; DAMP, damage-associated molecular pattern.
