# Supplementary figures and images for: Transcriptional and Histochemical Signatures of Bone Marrow Mononuclear Cell-Mediated Resolution of Synovitis
Source: Front Immunol. 2021 Dec 8;12:734322. doi: 10.3389/fimmu.2021.734322 (PMC8692379; doi:10.3389/fimmu.2021.734322)

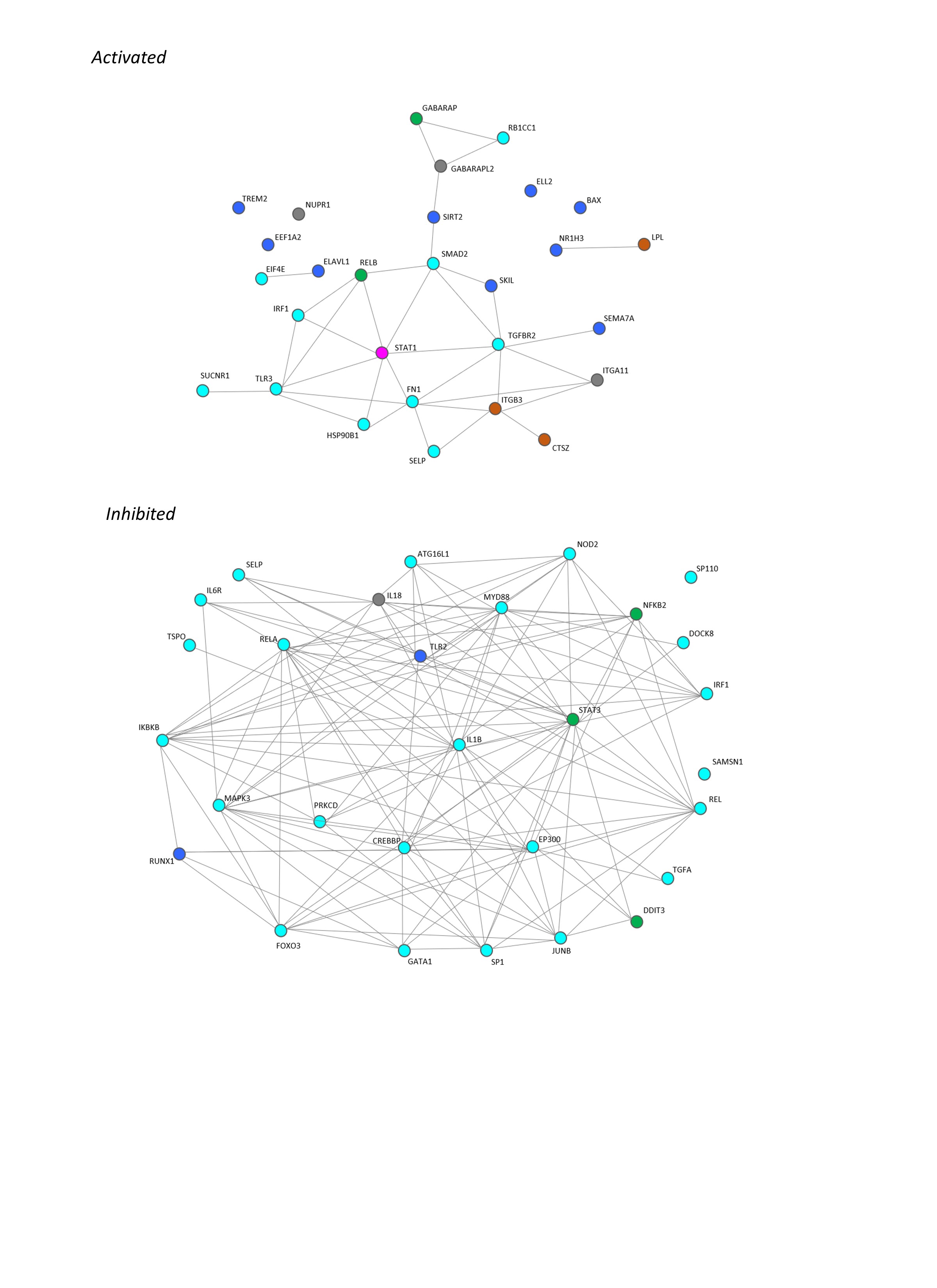

Supplement: Supplementary file 1 [file Image_1.jpg]
